# *Komagataella phaffii* as Emerging Model Organism in Fundamental Research

**DOI:** 10.3389/fmicb.2020.607028

**Published:** 2021-01-11

**Authors:** Lukas Bernauer, Astrid Radkohl, Leonie Gabriela Katharina Lehmayer, Anita Emmerstorfer-Augustin

**Affiliations:** ^1^Institute of Molecular Biotechnology, Graz University of Technology, NAWI Graz, BioTechMed-Graz, Graz, Austria; ^2^acib—Austrian Centre of Industrial Biotechnology, Graz, Austria

**Keywords:** *Pichia pastoris*, *Komagataella phaffii*, methylotrophic yeast, yeast genetics, mating, CRISPR/Cas9, chromosome structure, pexophagy

## Abstract

*Komagataella phaffii* (*Pichia pastoris*) is one of the most extensively applied yeast species in pharmaceutical and biotechnological industries, and, therefore, also called the biotech yeast. However, thanks to more advanced strain engineering techniques, it recently started to gain attention as model organism in fundamental research. So far, the most studied model yeast is its distant cousin, *Saccharomyces cerevisiae*. While these data are of great importance, they limit our knowledge to one organism only. Since the divergence of the two species 250 million years ago, *K. phaffii* appears to have evolved less rapidly than *S. cerevisiae*, which is why it remains more characteristic of the common ancient yeast ancestors and shares more features with metazoan cells. This makes *K. phaffii* a valuable model organism for research on eukaryotic molecular cell biology, a potential we are only beginning to fully exploit. As methylotrophic yeast, *K. phaffii* has the intriguing property of being able to efficiently assimilate methanol as a sole source of carbon and energy. Therefore, major efforts have been made using *K. phaffii* as model organism to study methanol assimilation, peroxisome biogenesis and pexophagy. Other research topics covered in this review range from yeast genetics including mating and sporulation behavior to other cellular processes such as protein secretion, lipid biosynthesis and cell wall biogenesis. In this review article, we compare data obtained from *K. phaffii* with *S. cerevisiae* and other yeasts whenever relevant, elucidate major differences, and, most importantly, highlight the big potential of using *K. phaffii* in fundamental research.

## Introduction: Why Use *K. phaffii* As Model Organism?

Most of the species that enabled the majority of biological discoveries during the last century did not start out as genetic model organisms, but rather matured into their roles. By being abundant human commensals (e.g., *Escherichia coli*, *Saccharomyces cerevisiae, Schizosaccharomyces pombe*, *Caenorhabditis elegans*, *Drosophila melanogaster*, and *Mus musculus*), they were easily available, and in addition found to be amenable to genetic modification. Among those model organisms, yeasts pose a special role. As unicellular organisms, they possess many of the advantages that made *E. coli* the first model organism for molecular biology, e.g., fast growth, cheap and easy cultivation conditions, as well as well-established and precise genetic modification strategies. The use of single celled organisms allows one to work with large numbers of individuals, e.g., to discover rare phenotypes and the genes involved in particular biological processes. The composition of the growth medium and the growth conditions can be varied quickly, whereas high-throughput screening assays can be applied easily. As simple eukaryotes, yeasts can be used to study processes that are conserved from yeast to humans, such as cell organelle biogenesis, cell cycle progression, cytoskeletal organization, DNA replication, and protein secretion. *S. cerevisiae* (commonly known as baker’s yeast, brewer’s yeast, or simply yeast) has long dominated yeast-based research. *S. cerevisiae* was the first eukaryotic organism to have its genome sequenced (Goffeau et al., 1996), and genetic manipulation techniques were established early on. Additionally, it has the ability to grow as a stable haploid or diploid, which facilitates the analysis of mating behavior and the characterization of essential genes. However, new advances in genetic modification techniques (e.g., CRISPR/Cas9) allow many more species to enter the privileged circle of “model organisms,” and other yeasts started to gain attention, e.g., *Kluyveromyces lactis*, *Kluyveromyces marxianus*, *Scheffersomyces stipitis*, *Yarrowia lipolytica*, *Arxula adeninivorans*, *Ogataea (Hansenula) polymorpha*, and, as will be highlighted in this review article, *Komagataella phaffii* (Löbs et al., 2017; Gündüz Ergün et al., 2019; Sibirny, 2019). While non-conventional yeasts have hitherto widely been used for a range of biotechnological applications, they are now starting to gain attention as valuable model systems in fundamental research.

*K. phaffii* (often still referred to by its obsolete name *Pichia pastoris*) is an obligate aerobic and methylotrophic yeast, which means that it can metabolize methanol as sole carbon and energy source. The first enzyme in the methanol assimilation pathway is alcohol oxidase (AOX). In *K. phaffii*, alcohol oxidase (AOX) is encoded by two genes, *AOX1* and *AOX2* (Cregg et al., 1989). The availability of the extremely strong *AOX1* promoter was one of the major reasons why *K. phaffii* gained attention as expression host for recombinant proteins in the first place. One of the most common strategies for (methanol-induced) gene expression is to integrate a recombinant gene into the *AOX1* region (Invitrogen, 2014). While it was proven to be an easy-to-target locus for gene insertions, the knockout of *AOX1* also creates a Mut^S^ strain. Mut^S^ strains still grow slowly on methanol due to the expression of *AOX2*, but direct the force of the *AOX1* promoter mainly toward recombinant protein production.

**FIGURE 1:**
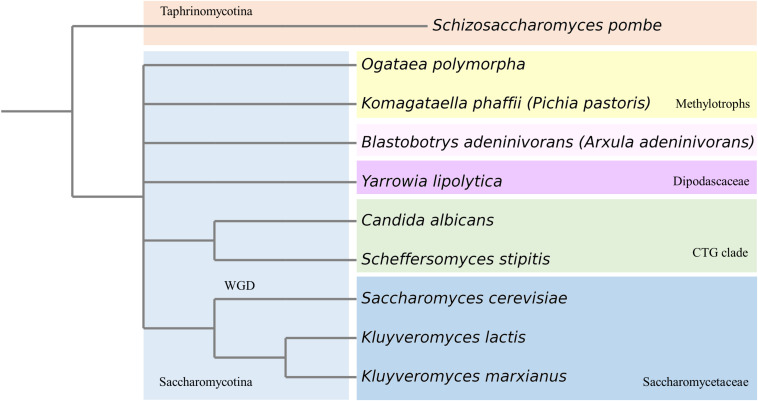
Phylogenetic tree of most prominent yeasts used in (fundamental) research. WGD, whole genome duplication.

Since *K. phaffii* became such a popular production host for the pharmaceutical, feed and food industries, it is also called the biotech yeast. *K. phaffii* has several advantages over *S. cerevisiae*, including better thermo- and osmo-tolerance, respiratory growth to extremely high cell densities, effective protein secretion, and the ability to express recombinant proteins at high levels from strong constitutive and inducible promoters (Karbalaei et al., 2020). Furthermore, secretory mammalian protein production has been facilitated upon extensive engineering of glycosylation pathways (Hamilton et al., 2003; Hamilton and Gerngross, 2007; Jacobs et al., 2009). Bioprocesses also benefit from *K. phaffii’*s ability to efficiently produce membrane proteins (Jahic et al., 2006; Byrne, 2015). While most of these advances served the primary purpose to improve heterologous protein production, they also created an explosion in the knowledge based on the system as described in numerous publications. This, in turn, inspired a significant number of research labs to start using *K. phaffii* for fundamental studies. As methylotrophic yeast, *K. phaffii* exhibits methanol assimilation pathways, which cannot be studied in yeasts that either did not evolve or lost this feature in the course of evolution (Riley et al., 2016). Phylogenetically, *Komagataella* species are members of the methylotrophic yeasts clade (family *Phaffomycetaceae*) and are only distantly related to better-known yeasts such as *S. cerevisiae, S. pombe*, and *Candida albicans* (Riley et al., 2016; Shen et al., 2018; Figure 1). This fact adds great value to *K. phaffii* as emerging model yeast. If a process is found to be conserved among *K. phaffii, S. cerevisiae*, and other yeasts, it is likely to be more widely conserved. *Vice versa*, mechanistic differences found between different yeasts may indicate functional diversity among higher eukaryotes (Table 1).

**TABLE 1:** Overview of the most prominent differences between *S. cerevisiae* and *K. phaffii.*

	*S. cerevisiae*	References	*K. phaffii*	References
Properties	Non-pathogenic, facultative anaerobic, Crabtree positive budding yeast		Non-pathogenic, obligate aerobic, Crabtree negative budding yeast	Heistinger et al., 2020
Genome size	12 Mbps	Goffeau et al., 1996	9.4 Mbps	De Schutter et al., 2009
Number of chromosomes	16 chromosomes (haploid cell)	Goffeau et al., 1996	4 chromosomes (haploid cell)	De Schutter et al., 2009
Type of centromeres	Point centromere	Hegemann and Fleig, 1993	IR centromere	Coughlan et al., 2016
Number of genes	5,885 potential protein-encoding genes; ∼450 genes for ribosomal RNA, small nuclear RNA, and transfer RNA genes	Goffeau et al., 1996	5,256 putative open reading frames in CBS7435, 5111 thereof manually curated	Sturmberger et al., 2016
Ploidy	Spontaneously changes ploidy, wild type isolates are mostly diploid	Knop, 2006; Harari et al., 2018	Most stable in the vegetative haploid state	Cregg, 1987
Whole genome duplication	Yes	Kellis et al., 2004	No	Valli et al., 2016
DNA repair mechanism	Mainly homologous recombination	Brachmann et al., 1998	Mainly non-homologous end-joining	Näätsaari et al., 2012
Mating behavior	Mating of heterothallic strains highly efficient (∼50%)	Schrick et al., 1997	Mating of heterothallic strains 100 times less efficient than in *S. cerevisiae* (∼0.5%)	Chen et al., 2012
Favored carbon source	Glucose	Kilkenny and Hinshelwood, 1951	Glucose, glycerol, methanol	Riley et al., 2016
Special growth requirements	Grows well in YPD and minimal media containing glucose	Alloue-Boraud et al., 2015	Grows well in YPD, but minimal media should be supplemented with biotin	Jungo et al., 2007; Invitrogen, 2014
Optical densities (OD_600_)	OD_600_ ∼ 50 in a bioreactor	Walther et al., 1996	OD_600_ ∼500 in a jar fermenter	Cereghino and Cregg, 2000
Hypoxic conditions	Glycolysis, pentose phosphate pathway and TCA cycle not massively affected	Daran-Lapujade et al., 2004; de Groot et al., 2007	Induction of glycolysis and the pentose phosphate pathway, downregulation of the TCA	Baumann et al., 2010
Osmoregulation	Glycerol is the main osmolyte released during hypo-osmotic shock	Nevoigt and Stahl, 1997	Arabitol is the main osmolyte released during hypo-osmotic shock	Kayingo et al., 2001
Macropexophagy	In cells transferred from oleate to glucose medium lacking a nitrogen source	Hutchins et al., 1999	In cells transferred from methanol to ethanol or from oleate to glucose medium	Nazarko et al., 2007
Structure of ER and Golgi apparatus	Entire ER network functions as transitional ER, Golgi exists as individual cisternae throughout the cytoplasm	Preuss et al., 1992; Rossanese et al., 1999	Discrete transitional ER sites and coherent Golgi stacks present in the cell	Rossanese et al., 1999; Bevis et al., 2002
Protein glycosylation	Golgi-resident α-1,3-mannosyltransferase present	Wiggins and Munro, 1998	Golgi-resident α-1,3-mannosyltransferase absent	Spadiut et al., 2014
Fatty acid compositions in membranes	Unsaturated and monounsaturated fatty acids	Stukey et al., 1989	Unsaturated, mono- and polyunsaturated fatty acids	Grillitsch et al., 2014
Lipid droplets	Triacylgylercol to steryl ester ratio ∼ 1:1	Grillitsch et al., 2011	Triacylgylercol to steryl ester ratio ∼ 15:1	Ivashov et al., 2012
Sphingolipids	(G)IPCs	Dickson, 2010	(G)IPCs and GlcCers	Ternes et al., 2011
Cell wall integrity sensors	Wsc1, Wsc2, Wsc3, Mid2, Mtl1	Levin, 2011	Wsc1, Wsc2, Wsc3	Ohsawa et al., 2017

## Origins of *K. phaffii* Research

**FIGURE 2:**
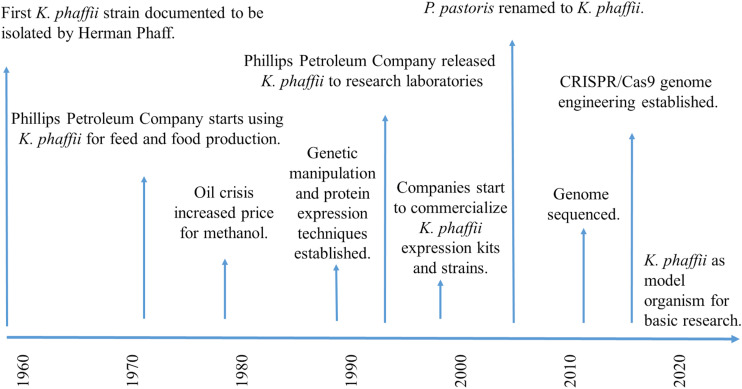
Timescale showing the major milestones of *K. phaffii* in research.

For decades, *K. phaffii* has been thought to belong to the genus *Pichia*, and therefore, mistakenly been called *Pichia pastoris*. The first strain ever assigned to the species *P. pastoris* was isolated by Alexandre Guilliermond from the exudate of a French chestnut tree in 1919, and was originally named *Zygosaccharomyces pastori* (species type strain CBS704, NRRL Y-1603) (Guilliermond, 1920; Figure 2). In the 1950s, Herman Phaff isolated further strains from black oak trees in California, United States, and renamed the species to *Pichia pastoris* (Phaff et al., 1956). However, in 1995, new insights generated by sequencing of ribosomal RNA caused all *P. pastoris* strains to be moved to a new genus, *Komagataella* (Yamada et al., 1995), and later separated into two species (Kurtzman, 2005): *Komagataella pastoris* (which includes the French strain) and *K. phaffii* (which includes the American isolates). The members of the genus *Komagataella* are phenotypically too similar to be distinguished from one another by routine tests usually employed in yeast taxonomy, which is why classification is mainly based on sequence alignments of a limited number of genes. The genomes of *K. pastoris* and *K. phaffii* differ by approximately 10% DNA sequence divergence and two reciprocal translocations (Love et al., 2016), and both strains are in use for recombinant protein production under the name *P. pastoris*. This seems quite confusing, but exact strain identifications, such as CBS2612 (NRRL Y-7556) or CBS7435, usually help allocate which (wild type) strain has been used. At this point it should be noted, that applied sciences still preferentially use the name *Pichia pastoris* (often as a synonym for all *Komagataella* species), while fundamental studies rather shifted to using the correct name, *K. phaffii.*

On a commercial basis, *K. phaffii* initially gained attention for the production of biomass and single-cell protein from methanol, a back then comparably cheap carbon source. In the 1970s, Phillips Petroleum Company (Bartlesville, Okla, United States), [and continued by Research Corporation Technologies (RCT)] started to develop and improve *K. phaffii* (CBS7435) fermentations for industrial production of high protein animal feed and biomass (Wegner, 1983, 1990). However, the world-wide oil crisis increased the price for methanol, and the economics of biomass production became unfavorable compared to proteins extracted from soybean and other sources. Phillips Petroleum Company decided to release the expression system to research laboratories in 1993 (BioWorld^TM^, 1993), and in the following years, diverse academic groups and biotech companies started to shift their interest toward the exploitation of *K. phaffii* as potential host for recombinant protein production (Wegner and Harder, 1987; Cereghino and Cregg, 2000). In the 1990s, a diverse set of expression vectors and genetic strain modification techniques started to be developed, published and commercialized (reviewed by Ahmad et al., 2014). Invitrogen (now Thermo Fisher Scientific, Waltham, Massachusetts, United States), for example, put their well-known “*Pichia* expression Kit” to market, containing two expression strains (GS115 and KM71), two different expression vectors, either with (pPIC9) or without (pHIL-S1) a secretion signal, for stable and selectable insertion of any gene of interest into the *AOX1* gene locus, as well as extensive and easy to follow method descriptions (Invitrogen, 2014). The “*Pichia* Expression Kit” has been used by many laboratories world-wide to get started with protein expression in *K. phaffii*. Initially in collaboration with the Glieder group, VTU Technology (now Validogen GmbH, Graz, Austria), generated a set of methanol-inducible and methanol-free *AOX1* promoter libraries with varying strengths, as well as diverse platform strains, which they now commercialize under the name “UNLOCK *PICHIA*”^1^. Simultaneously, many renowned laboratories published excellent “freedom to operate” studies on expression systems and genome modification tools, e.g., the Cregg, Glieder, Matthanovich, Schwab, Lin-Cereghino, Callewaert, and Ferrer groups. In this regard, we would like to highlight that close collaboration of research groups like the Mattanovich, Callewaert, and the Glieder group with industrial partners had a strong impact on the industrial application and commercialization of *K. phaffii*. Additionally, many of these research labs and companies (e.g., Bisy GmbH, Biogrammatics Inc., Bioingenium, Research Corporation Technologies (RCT) and Validogen GmbH) provide a diverse set of engineered *K. phaffii* strains, e.g., displaying improved protein expression, secretion, and glycosylation. On a technical basis, hands-on workshops have proven to be helpful to experience in person how to handle and genetically modify *K. phaffii* (e.g., https://www.hands-on-pichia.com/).

Even though the many strategies for recombinant protein expression in *K. phaffii* are highly interesting and constantly evolve (Ahmad et al., 2019), they are beyond the focus of this review, which is why we would like to refer the reader to some of the many excellent review articles summarizing and describing the progress made in this field (Ahmad et al., 2014; Karbalaei et al., 2020).

## *K. phaffii* Genome Content and Chromosome Organization

Even though many *K. phaffii* strains have been isolated from methanol enriched media since the 1960s (Ogata et al., 1969; Kato et al., 1974), only four distinct natural isolates of *K. phaffii* exist in public yeast culture collections (Braun-Galleani et al., 2019). To date, almost all laboratory work on *K. phaffii* utilizes strains derived from the same natural isolate, CBS7435 (Braun-Galleani et al., 2019). For example, *K. phaffii* CBS7435 is the parental strain of the broadly commercialized GS115, which was developed by RCT/Philips petroleum by chemical mutagenesis and selected for histidine auxotrophy (US Patent 4,879,231 A). One of the biggest milestones in *K. phaffii* related research was the whole-genome sequencing of important *K. phaffii* strains GS115 (De Schutter et al., 2009) and CBS7435 (Küberl et al., 2011; Sturmberger et al., 2016), and *K. pastoris strain* DSMZ 70382 (Mattanovich et al., 2009). The genome sequences provide a robust platform for the ongoing annotation of features and functional annotations of those features to the genome content. To facilitate communication, the genetic nomenclature used for *S. cerevisiae* has been applied to *K. phaffii* (Valli et al., 2016). For example, the *HIS4* gene in both *S. cerevisiae* and *K. phaffii* encodes histidinol dehydrogenase. There is also cross-complementation between gene products in both *S. cerevisiae* and *K. phaffii*. Several wild type genes from *K. phaffii* complement comparable mutant genes (e.g., *HIS4, ARG4, TRP1, URA3*, and *ADE1*) in *S. cerevisiae* (Cosano et al., 1998; Lin Cereghino et al., 2001). Gradually, a more complete picture of *K. phaffii* is emerging as new data sets are collected from a diverse range of experiments to refine the annotated gene structures and assign functional data to them. For example, in order to identify essential genes in *K. phaffii*, a combination of transposon mutagenesis and high-throughput sequencing has been applied (Zhu et al., 2018), and based on the genome sequencing of *K. phaffii*, genome-scale metabolic networks could be reconstructed to model the yeasts’ metabolism from a systems perspective (reviewed by Chung et al., 2013).

The *K. phaffii* genome size is 9.4 Mbp, compared to the 12 Mbp genome of *S. cerevisiae*. Despite similar genome sizes, *K. phaffii* has only four relatively large chromosomes of 2.9, 2.4, 2.3, and 1.8 Mbp in contrast to 16 smaller chromosomes in *S. cerevisiae*. With an average size of 2.5 Mbp, *K. phaffii* chromosomes are almost twice the length of the largest *S. cerevisiae* chromosome (chromosome I), which is 1.5 Mbp. Recently, Coughlan et al. structurally defined the centromeres of *K. phaffii* by transcriptome sequencing (RNA-seq) and chromatin immunoprecipitation with high-throughput sequencing (ChIP-seq) by binding the histone H3-like centromere protein Cse4 (Coughlan et al., 2016). This study demonstrates that *K. phaffii* has large modular centromeres, more reminiscent of those of higher organisms than of the 125 bp element sufficient for centromere function in *S. cerevisiae* (Fitzgerald-Hayes et al., 1982). Its four centromeres are unrelated in sequence and consist of a 1 kb central core (mid) region flanked by a 2 kb inverted repeat (IR). The histone H3 variant CenH3 (Cse4) binds strongly to the mid region, and gradually less strongly along the IRs. In principle, the organization and structure of *K. phaffii* centromeres resembles those of *S. pombe* (Wood et al., 2002), but they are smaller in size and lack the extensive flanking heterochromatic outer repeats. Due to recombination in the IRs, different isolates of *K. phaffii* show polymorphism for the orientation of the mid regions. The general conservation of centromere features, including size, structure, and multilayered organization, highlights *K. phaffii* as a valuable model for the study of eukaryotic chromatin remodeling and centromere function.

## Growth Behavior: Carbon Sources, Methanol Assimilation, and Auxotrophies

In principle, *K. phaffii* can be grown on the same media as *S. cerevisiae*, e.g., YPD (yeast extract, peptone dextrose/glucose) and minimal media containing a nitrogen source and glucose as main carbon source. In these media, the doubling time of a log phase wild-type strain is ∼2 h. However, it has been shown that *K. phaffii* has a special requirement for biotin, which is why there have been efforts to optimize biotin concentrations in bioreactor cultivations (Jungo et al., 2007), or to engineer strains to become prototrophic for biotin (Gasser et al., 2010). Based on transciptomics, the Love lab created a specially designed rich medium for large-scale cultivations (Matthews et al., 2018). *K. phaffii* can utilize glucose, glycerol, sorbitol, methanol, ethanol, L-rhamnose, and acetate as carbon source (Riley et al., 2016). A comprehensive overview of the carbon sources and their diauxism is given by Zepeda et al. (2018).

As most yeasts, *K. phaffii* is “Crabtree-negative,” which means that it produces energy mostly via respiration. Oxygen limitation forces a cell to readjust its metabolic fluxes from cellular respiration to fermentation, which causes massive energy deprivation in a cell due to strongly reduced availability of ATP. Therefore, *K. phaffii* is more sensitive to the availability of oxygen than the “Crabtree-positive” yeast *S. cerevisiae* (Pfeiffer and Morley, 2014). There are several discussions, why and how a small set of yeasts evolved Crabtree positive phenotypes. Clearly, it gives them an evolutionary advantage of consuming glucose faster and producing ethanol to outcompete other microorganisms in sugar rich environments. The Mattanovich lab recently managed to convert *K. phaffii* into a Crabtree positive yeast through overexpression of a single Gal4-like transcription factor, which provides novel insights into the evolution of the Crabtree effect (Ata et al., 2018). As obligate aerobe, *K. phaffii* displays exclusively respiratory metabolism and naturally does not switch to an anaerobic metabolism that would lead to toxic metabolite accumulation under oxygen limited conditions. This is the main reason why such high cell density fermentations can be achieved with this organism under dissolved oxygen controlled processes. Another interesting engineering approach recently published converted *K. phaffii* into an autotroph that grows on CO_2_. In this study, the insertion of eight heterologous genes and deletion of three native genes generated a CO_2_-fixation pathway resembling the Calvin–Benson–Bassham cycle, the predominant natural CO_2_-fixation pathway (Gassler et al., 2020). Adaptive laboratory evolution could improve the growth rate from 0.008 to 0.018 h^–1^.

As methylotrophic yeast, *K phaffii* is able to grow on methanol as sole carbon source. This makes it one of approximately a dozen yeast species representing four different genera capable of assimilating methanol. The other genera include *Candida, Ogataea*, and *Torulopsis*. The methanol utilization (MUT) metabolic pathway appears to be the same in all these yeasts and involves a unique set of pathway enzymes—including alcohol oxidase (AOX), dihydroxyacetone synthase (DAS), and formate dehydrogenase (FDH) (Hartner and Glieder, 2006; Yurimoto and Sakai, 2009). In *K. phaffii*, the MUT pathway has been studied extensively, which is summarized in some excellent review articles (Daly and Hearn, 2005; Hartner and Glieder, 2006). Therefore, we will just outline the most important key aspects of methanol utilization. The first step of the MUT pathway is the oxidation of methanol to formaldehyde and hydrogen peroxide in the peroxisomes by alcohol oxidase (AOX). In *K. phaffii*, AOX is encoded by two genes, *AOX1* and *AOX2* (Cregg et al., 1989). Methanol assimilation is subject to carbon-source-dependent repression, derepression, and induction mechanisms. The presence of glucose, glycerol, and ethanol represses expression of both AOX genes, whereas the presence of methanol strongly induces expression at non-growth-limiting conditions (reviewed by Vogl and Glieder, 2013). Certain growth conditions can also trigger co-assimilation of a multicarbon source and methanol (Egli et al., 1982). Although glycerol does not derepress expression of *AOX1*, other non-repressing carbon sources like sorbitol, mannitol, trehalose, and alanine do so (Thorpe et al., 1999; Inan and Meagher, 2001). Aox1 has a much higher cellular abundance than Aox2. Thus, knockout of *AOX2* results in a wild type like phenotype on methanol (Mut^+^), while an *AOX1* knockout exhibits extremely slow growth on methanol (Mut^S^, methanol utilization slow). Double knockout strains are unable to grow on methanol (Mut^–^). After the oxidation of methanol to formaldehyde and hydrogen peroxide, catalase breaks down the toxic hydrogen peroxide to water and oxygen. Formaldehyde can either undergo a dissimilation pathway by two subsequent dehydrogenase reactions or an assimilation pathway by condensation with xylulose 5-phosphate (Xu5P). A dihydroxyacetone synthase (DAS) catalyzes the latter peroxisomal condensation reactions of Xu5P and formaldehyde into the C3-compounds dihydroxyacetone (DHA) and glyceraldehyde 3-phosphate (GAP), which are further metabolized in the cytosol. Again, all of these steps are explained in much detail by Hartner and Glieder (2006).

In the last decades, metabolic studies and the generation of genome-scale metabolic models have been successfully employed to characterize the cellular physiology of *K. phaffii* and improve metabolic engineering (reviewed by Chung et al., 2013). In general, comparative genomics enabled the direct comparison of different yeast species and shows their differences, e.g., growth behavior under certain conditions on a broad basis (Solà et al., 2007; Dragosits et al., 2009). Chung et al. (2010) highlight and compare three genome-scale metabolic models available for *K. phaffii* and *Pichia stipitis* (Chung et al., 2010; Sohn et al., 2010; Caspeta et al., 2012). Overall, diverse metabolic pathways have been constructed and the metabolites and reactions assigned to subcellular compartments including the cytosol, endoplasmic reticulum, extracellular fluid, Golgi apparatus, mitochondria, nucleus, peroxisome, and vacuole. Growth predictions made on the basis of these models came reasonably close to the experimental data reported in earlier studies. In another interesting, recent study, organelles were isolated from *K. phaffii* cells grown on glucose or methanol, the proteome quantified and compared to *S. cerevisiae* (Valli et al., 2020). Differences in protein localization were found mostly for cytosolic, mitochondrial, and peroxisomal proteins, and the extensive analysis of the carbon-source dependent, organelle specific proteome gives insight in protein localization on a very broad basis. Both strategies, genome-scale metabolic models and the subcellular proteome atlas, provide powerful tools for targeted strain engineering strategies.

## Genetic Manipulation Techniques

Based on the vast amount of excellent reviews on this topic (Cereghino and Cregg, 2000; Ahmad et al., 2014; Baghban et al., 2018, to name a few), we will keep this chapter rather short, and only focus on the most relevant aspects for the present review article.

### Autonomously Replicating Plasmids

In many yeasts, as for example in *S. cerevisiae* and *S. pombe*, gene expression can be driven from small extrachromosomal plasmids (Siam et al., 2004; Gnügge and Rudolf, 2017). The use of plasmids has the big advantage that they can be cloned and transformed easily, but also get rid of, if not required any longer. Depending on the type of origin of replication, cells can either contain one (CEN/ARS origin) or several (two micron circle replication origin) copies of the plasmid, which can help modulate protein production levels. Autonomously replicating sequences (ARSs), which generally serve as the origins of DNA replication during mitosis, constitute the primary origins utilized to replicate plasmids in yeast host cells. A *K. phaffii*-specific ARS (*PARS1*) was identified over 30 years ago (Cregg et al., 1985). Similar to the *S. cerevisiae*-specific ARS, *PARS1* enabled the high-efficiency transformation of *K. phaffii* with circular plasmids (Weninger et al., 2016; Pan et al., 2018). Recently, a 452 bp ARS element (panARS) was identified in *K. lactis* and shown to facilitate transformation in a wide range of yeast species, including *K. phaffii* (Liachko and Dunham, 2014; Camattari et al., 2016), and a mitochondrial DNA (mtDNA) fragment was discovered to function as a novel ARS in *K. phaffii* (Schwarzhans et al., 2017). All plasmids bearing these ARS sequences were found to be poorly stable for replication and segregation in *K. phaffii*, which restricted their use in many applications. Plasmid instabilities are not only detrimental for recombinant protein expression, but may also lead to unwanted random integration of the plasmid DNA into the yeast genome, especially if selection pressure is applied. As discussed earlier, recent advances in identifying putative centromeres on each of the four *K. phaffii* chromosomes (Coughlan et al., 2016; Sturmberger et al., 2016) now enabled the construction of more stably replicating episomal plasmids containing the entire chromosome two centromere DNA sequence (Nakamura et al., 2018).

However, for some applications it is beneficial to apply plasmids with low stability. For example, in CRISPR/Cas9 applications, it is important for cells to lose Cas9 activity after cell engineering to avoid unspecific mutations in the genome. To that effect, Weninger et al. (2016, 2018) successfully used PARS1 in their CRISPR/Cas9 plasmids, whereas a more recent study from Gu et al. (2019) compared different ARS sequences in CRISPR/Cas9 approaches and found very good efficiencies with plasmids containing panARS. In order to get rid of the plasmid after genome engineering, cells simply need to be streaked out on plates without selection pressure. Single colonies grown on these plates have usually already lost the plasmid.

### DNA Recombination

Genomic integration of expression cassettes and DNA fragments can occur via two distinct DNA repair mechanisms in eukaryotic cells: homologous recombination (HR) and non-homologous end joining (NHEJ). HR is a highly accurate repair mechanism mediated through base pairing of rather long stretches of homologous DNA sequences and catalyzed by proteins encoded by genes in the *RAD52* epistasis group (Pastwa and Blasiak, 2003). In contrast, NHEJ requires no sequence homology to operate (Krejci et al., 2012). The heterodimer Ku70/80 binds to free ends of a DNA strand and recruits DNA protein kinases (DNA-PKCs) to initiate a quick and unspecific repair of the DNA double strand break (Pastwa and Blasiak, 2003; Dudásová et al., 2004). Both mechanisms play overlapping roles in yeasts, but are used to a different extent depending on the yeast species. In *S. cerevisiae*, HR represents the dominant repair mechanism, whereas NHEJ occurs very rarely. In order to achieve targeted gene knockouts and knockins, short homologous flanking regions of 40 bp were shown to be sufficient (Brachmann et al., 1998), and integration events usually occur with > 70% efficiency at the correct locus (Gueldener et al., 2002). This highly efficient mechanism offers another intriguing possibility; a process known as *in vivo* ligation, which promotes the direct self-assembly of multiple recombinant DNA fragments in the nucleus of *S. cerevisiae* and fully eliminates the cloning process for plasmid assembly (Oldenburg et al., 1997; Juhas and Ajioka, 2017). *In vivo* recombination could also be observed in *K. phaffii* when a library of *Rhizopus chinensis* lipase mutants was assembled directly by the host and integrated into the targeted genomic locus (Yu et al., 2012). Overlapping ends as short as 50 nucleotides were reported to be sufficient to promote assembly at a relatively high efficiency. However, this seems rather surprising, since recombination and targeted gene insertions were shown to be difficult to achieve in *K. phaffii* because of the high ratio of NHEJ-to-HR activity (Näätsaari et al., 2012; Tsakraklides et al., 2015; Schwarzhans et al., 2016). For example, homologous targeting sequences of < 500 bp only led to < 0.1% of positive targeting events indicating a very low efficiency of gene replacement. This efficiency could be increased up to 1.5-fold by extending the homologous regions up to 1 kb at each side (Näätsaari et al., 2012). Due to this low efficiency, several strategies were developed to improve HR activity in *K. phaffii*, e.g., hydroxyurea-mediated cell cycle arrest (Tsakraklides et al., 2015) and providing extra copies of the gene to be deleted on a helper plasmid (Chen et al., 2013). Deletion of *KU70* also significantly improved the NHEJ-to-HR ratio (Näätsaari et al., 2012). Deletion of *KU70* can, however, also negatively affect cellular fitness (Näätsaari et al., 2012). This has also been demonstrated indirectly in a study where a CBS7435 *ku70*Δ was engineered toward production of different terpenoids (Wriessnegger et al., 2015). RNAseq data revealed that *RAD52*, a protein involved in DNA repair, was highly upregulated in all of the *ku70* deletion strains. Methanol-induced overexpression of *RAD52* drastically improved terpenoid production, which was most probably due to rescuing the loss of Ku70 function. In contrast, *RAD52* overexpression had no effect on a *S. cerevisiae* strains producing terpenoids.

HR frequency can also be increased by several orders of magnitude (up to 4,000-fold in *S. cerevisiae*) through targeted single and double strand break induced DNA repair (Storici et al., 2003; Caldecott, 2008), which is why precise mechanisms introducing double-strand breaks in the genome are of great interest. The currently most prominent system that mediates targeted genome engineering in various pro- and eukaryotic hosts is CRISPR/Cas9 (Jakočiunas et al., 2016; Stovicek et al., 2017; Adli, 2018). The CRISPR gene-editing technology is composed of an endonuclease protein (Cas9), whose DNA-targeting specificity and cutting activity can be programmed by a short guide RNA (sgRNA). Optimal conditions required for efficient CRISPR/Cas9 function are very narrow in *K. phaffii*. Weninger et al. (2016) systematically tested more than 90 constructs containing different codon optimized DNA sequences of *CAS9*, various sgRNA sequences, several RNA Pol III, and RNA Pol II promoters (in combination with ribozymes) for the expression of the sgRNAs and different RNA Pol II promoters for the expression of *CAS9* and sgRNAs. Only ∼6% (6/95) of all tested constructs mediated efficient CRISPR/Cas9 targeting, namely those bearing RNA Pol II promoters, ribozymes, and a human codon optimized *CAS9* sequence. Multiplexity could be proven upon expressing different sgRNAs targeting multiple loci from one plasmid (Weninger et al., 2016; Liu et al., 2019). Follow-up studies have shown that deletion of *KU70* drastically improved the efficiency of CRISPR/Cas9 engineering approaches due to the downregulation of NHEJ (Weninger et al., 2018; Liu et al., 2019).

*K phaffii*’s preference for NHEJ can also be advantageous for the construction of random gene knockout or knockin libraries. In *S. cerevisiae*, which prefers HR over NHEJ, this can only be achieved by labor intense implementation of transposon insertion strategies (Xu et al., 2011) or yeast oligo-mediated genome engineering (YOGE, a recombineering strategy) (Dicarlo et al., 2013). Recently, we also published a broad set of auxotrophic *K. phaffii* strains in the CBS7435 strain background, including strains auxotrophic for arginine and lysine, but also novel strains, e.g., auxotrophic for proline (Ahmad et al., 2019). These strains broaden the spectrum of possible markers to be used and can be highly beneficial for future strain modification strategies.

## Mating, Sporulation, and Tetrad Dissection

The first step of sexual reproduction and mating in yeasts involves the mutual recognition of haploid cells of opposite mating types (*MAT***a** and *MAT*α). The expression of the respective *MAT* gene hence determines the mating type of a cell, and the process causing DNA rearrangements in sexually differentiating cells is called mating type switching (Hanson and Wolfe, 2017). In *S. cerevisiae*, mating type switching is enabled through the presence of silent copies of both *MAT* variants, *HML*α and *HMR***a**. During mating type switching, HO endonucleases create double strand breaks at the *MAT* loci and genes at the active locus are replaced with a silent copy of the opposing mating type through synthesis dependent strand annealing (Haber, 2012). By definition, primary homothallic species usually express both *MAT* genes allowing them to mate with one other. Heterothallic species have individuals that reside in different mating types, and only cells of opposite mating types can mate. The same is true for secondary homothallic species, but cells can switch their mating types and mate with cells of the same strain. Since wild type *S. cerevisiae* strains are self-fertile, they are classified as secondary homothallic. Laboratory strains, which carry non-functional HO endonucleases are classified as heterothallic.

Mating type switching greatly differs between different yeast species, but has similar mechanisms in the methylotrophic yeasts *O. polymorpha* (Maekawa and Kaneko, 2014) and *K. phaffii* (Hanson et al., 2014). Both yeasts contain one copy each of *MAT***a** and *MAT*α. The loci are flanked by sequences containing inverted repeats that are orthologous to regions generally found in Saccharomycetaceae. In both yeasts, one of the *MAT* loci is located close to a heterochromatin region (a centromere in case of *O. polymorpha*, and a telomere in case of *K. phaffii*), which causes transcriptional repression of the respective *MAT* locus. The inverted repeat sequences flanking the *MAT* loci are essential for mating type switching. Mating type switching occurs by homologous recombination of these sequences, which inverts the entire genomic region between the *MAT***a** and *MAT*α locus (19 kb in *O. polymorpha*, 138 kb in *K. phaffii*). This means that once mating type switching is induced, active and repressed positions of *MAT* genes are swapped, without the involvement of any major synthesis or degradation of DNA. By contrast, in *S. cerevisiae* and *S. pombe* exonucleases degrade the exchanged *MAT* genes that have been replaced by newly synthesized DNA copied from the silent locus.

In contrast to *S. cerevisiae, K. phaffii* is most stable in the vegetative haploid state and (like *S. pombe* and *K. lactis*) remains haploid unless forced to mate, under certain conditions such as nitrogen limited-starvation (Cregg, 1987). Nitrogen limitation and other nutritional stresses also cause mated diploid *K. phaffii* cells to efficiently undergo meiosis, sporulation, and rapidly switch back to the haploid state. Upon mating two *K. phaffii* strains expressing the heavy and the light chain of the anti-HER2 antibody, it was shown that the mating efficiency of wild-type haploid *K. phaffii* strain (NRRL-Y11430) is about 0.1–1% (1 diploid per 1,000–100 haploid cells) (Chen et al., 2012). *S. cerevisiae*, in contrast, exhibits mating efficiencies of about 50% (Schrick et al., 1997).

The Mattanovich group recently published their studies on how to produce heterothallic *K. phaffii* strains with defined mating types (Heistinger et al., 2017, 2018). Even though tetrads appeared to be significantly less stable than described for *S. cerevisiae*, strains of opposite mating types were shown to efficiently mate and sporulate. The generation of heterothallic *S. cerevisiae* strains was one of the reasons for the establishment of this yeast as the most common yeast model organism. Hence, the establishment of heterothallic *K. phaffii* strains can be a powerful tool in *K. phaffii* related research. Heterothallic (or haploid) yeast cells are especially useful when screening for mutant alleles that produce a desired phenotype. Moreover, mating of *S. cerevisiae* has been successfully employed in diverse research applications such as yeast two-hybrid libraries (Golemis and Khazak, 1997). Also, the use of haploid cells of opposite mating types could greatly benefit the identification of synthetic lethality and facilitate complex strain construction, a task still difficult to achieve with *K. phaffii*.

## Peroxisome Studies: Proliferation and Pexophagy

Peroxisomes are essential, subcellular organelles that are ubiquitously present in all eukaryotic cells. They have the intriguing ability to sequester specific enzymes that metabolize a variety of substrates, which often enables an organism to survive in unique environments. For example, in *K. phaffii* and other methylotrophic yeasts, the metabolism of methanol takes place within the peroxisome in order to separate toxic hydrogen peroxide, one of the major catabolites of methanol utilization, from the rest of the cell (van der Klei et al., 2006). In methylotrophic yeasts, peroxisomal proliferation can easily be stimulated upon shifting cells to methanol-rich media (Yurimoto et al., 2011). *Vice versa*, the transfer of cells from methanol-rich to methanol-deplete media induces pexophagy, the selective autophagy of peroxisomes. This feature has made methylotrophic yeasts like *K. phaffii* an attractive model system to dissect the molecular mechanisms controlling peroxisome biogenesis, proliferation and degradation.

Peroxisomes proliferate either by growth and division of pre-existing peroxisomes or arise *de novo*. This knowledge has been substantiated by studies using yeast peroxisome-deficient mutants (pex mutants). The endoplasmic reticulum (ER) was shown to provide most of the lipids and proteins needed for the formation of peroxisomes. This has been indicated upon studying the trafficking of diverse peroxisomal membrane proteins (PMPs) in *S. cerevisiae, K. phaffii*, and *O. polymorpha*, e.g., Pex3, Pex19, and Pex22, which are delivered to the peroxisome through the ER (reviewed by Agrawal and Subramani, 2016; Yuan et al., 2016). PEX genes whose deletion results in abnormal peroxisome number and size are usually less well studied, because mutants defective in these genes often do not exhibit defective peroxisome function or sorting of PMPs. Interestingly, the phenotype of such mutants can vary from organism to organism. For example, the deletion of *PEX30* results in fewer and clustered peroxisomes in *K. phaffii*, while in *S. cerevisiae*, an increase in normal-sized peroxisomes has been reported (Yuan et al., 2016).

Upon glucose- or ethanol-induced catabolite inactivation, peroxisomes are rapidly and selectively degraded within the vacuole by a process called pexophagy, an autophagy-like process. In *K. phaffii*, pexophagy can proceed by micro or macro events (extensively reviewed by Dunn et al., 2005; Oku and Sakai, 2016). Micropexophagy is induced when methanol-grown cells are adapting to glucose, whereas macropexophagy is induced when cells are shifted from methanol to ethanol. During micropexophagy, an autophagic membrane structure called the micropexophagy-specific apparatus (MIPA) emerges on the peroxisome surface and fuses with arm-like extensions of the vacuole, which eventually engulfs the target peroxisome cluster (Mukaiyama et al., 2004). Although this mode of pexophagy has been extensively characterized in *K. phaffii*, similar dynamics of vacuole engulfment of the peroxisome were detected in oleate-grown *S. cerevisiae* cells when replenished with glucose (Chiang et al., 1996). During macropexophagy, the peroxisome is completely entrapped by a double-membrane structure, termed the macropexophagosome (MPP). The outer membrane of the MPP fuses with the vacuolar membrane and releases the captured peroxisome into the vacuolar lumen, a process that shows high similarity to macroautophagy. Macropexophagy has also been discovered in other yeast species, including *O. polymorpha* (when transferred from methanol to various other media) (Monastryska et al., 2004) and *S. cerevisiae* (when transferred from oleate medium to glucose medium lacking a nitrogen source) (Hutchins et al., 1999). Due to the vast amount of literature covering pexophagy in *K. phaffii*, we would like to refer the interested reader to excellent review articles published by the Veenhuis, Suzuki, and Sakai labs (Mukaiyama et al., 2004; Dunn et al., 2005; Oku and Sakai, 2016).

## Protein Secretion and Unfolded Protein Response

Yeast has always been an important model organism to study secretory events because of the high conservation of the secretory machinery from lower to higher eukaryotes. The yeast secretory pathway is a very complex process that involves about 160 proteins responsible for different post translational processes, such as glycosylation and folding (Nielsen, 2013). Among the 550 proteins, which carry signal peptides and are processed by the secretory pathway, only very few are secreted to the extracellular matrix. The majority is targeted to the endoplasmic reticulum (ER), Golgi, vacuole, and cytoplasm. All of these secretory proteins must be folded correctly in the ER, and any accumulation of misfolded proteins causes ER stress that induces unfolded protein response (UPR). Activation of the UPR results in transcriptional change of about 400 genes (Tyo et al., 2012) and upregulation of chaperones and foldases, as well as ER associated degradation (ERAD), whereas many of these processes are under the regulation of the Hac1p transcription factor. In contrast to *S. cerevisiae, K. phaffii* has discrete transitional ER sites (Bevis et al., 2002) and coherent Golgi stacks (Rossanese et al., 1999), which makes this yeast an ideal host for studying the organization of ER subdomains and the early secretory pathway. Similar to the studies done with *S. cerevisiae* by the Schekman lab (Novick et al., 1981), the Glick lab investigated *K. phaffii* homologs of the *S. cerevisiae SEC12, SEC13, SEC17, SEC18*, and *SAR1* genes and showed that they complement the corresponding *S. cerevisiae* mutants (Payne et al., 2000). Comparison of these proteins revealed differences in intron structures and conserved domains.

One of the biggest advantages of *K. phaffii* as industrial production host is the extraordinary efficiency of its protein secretion machinery (Damasceno et al., 2012; Puxbaum et al., 2015). Because *K. phaffii* secretes few of its own proteins, the secreted recombinant protein is usually the major polypeptide species found in the extracellular medium. The most extensively applied system is secretory expression of recombinant genes fused to the α-factor prepro leader sequence originating from *S. cerevisiae* (Fuller et al., 1988). Fusion proteins efficiently enter the secretory pathway, and mature in the late Golgi upon being processed by the *K. phaffii* endogenous Ca^2+^ dependent serine endoprotease Kex2. Several studies have proven that Kex2 activity is a rate limiting step in α-factor prepro leader sequence driven protein secretion in *S. cerevisiae* (Bevan et al., 1998) and *K. phaffii* (Yang et al., 2013; Sun et al., 2019).

The endeavor to optimize protein secretion also led to extensive studies of the UPR in *K. phaffii* (Gasser et al., 2007; Graf et al., 2008; Lin et al., 2013). In order to induce UPR, either *HAC1* was overexpressed or cells were treated with DTT, and microarray analysis was performed. Results were allocated to GO groups and compared to studies using *S. cerevisiae*. *K. phaffii* reacted to DTT treatment mainly by the regulation of genes related to chemical stimulus, electron transport, and respiration. In contrast, overexpression of *HAC1* induced many genes involved in translation, ribosome biogenesis, and organelle biosynthesis. This indicates that the cellular reactions to DTT treatment only slightly overlap with the reactions to overexpression of *HAC1*. In another study, the secretion of Fap fragments was examined under different oxygen conditions (Baumann et al., 2010). Gene expression profiling was combined with proteomic analyses and the ^13^C isotope labeling based experimental determination of metabolic fluxes in the central carbon metabolism. Since the protein expression machinery is a multistep metabolic process that requires ATP, it is not surprising that a shift to fermentative metabolism negatively impacted protein synthesis and secretion. However, more importantly, differences in the regulation of the core metabolism regulated by oxygen availability between *K. phaffii* and *S. cerevisiae* could be investigated. In contrast to studies using *S. cerevisiae* (de Groot et al., 2007), a strong transcriptional induction of glycolysis and the non-oxidative pentose phosphate pathways, as well as downregulation of the TCA cycle could be observed in *K. phaffii* under hypoxic conditions. Upon testing the influence of high osmolarity stress on protein secretion it has been demonstrated that processes such as protein folding, ribosome biogenesis and cell wall organization were affected (Dragosits et al., 2010). In contrast to *S. cerevisiae*, the main osmolyte released during hypo-osmotic stress was arabitol rather than glycerol, a response mainly observed in osmotolerant yeasts (Kayingo et al., 2001). Also, in contrast to *S. cerevisiae*, no transcriptional activation of the high osmolarity glycerol (HOG) pathway was observed at steady state conditions.

Another big, commercial research field is glycoengineering of *K. phaffii* for secretory expression of humanized glycoproteins. Due to its endogenous glycosylation machinery lacking specific terminal mannose glycopeptides known to be clinically incompatible for human administration (Hamilton and Gerngross, 2007), studies of antibody production in yeast have predominantly been conducted in *K. phaffii* (Spadiut et al., 2014). Several glycoengineering approaches provided valuable insights into the glycosylation machinery of *K. phaffii* and enabled the production of proteins with human linked glycans causing only mild to low antigenicity in humans (reviewed by Ferreira et al., 2019).

## Biological Membranes

Biological membranes play many essential roles. Most importantly, they serve as intracellular boundaries, which compartmentalize organelles and shield the interior of the cell from the exterior, but they also create an indispensable environment for membrane-linked enzymes and biosynthetic processes. Among other yeasts, *K. phaffii* is an interesting model to investigate diverse aspects of cellular membranes, e.g., lipid profiling, lipid storage and cellular responses caused by lipid modifications (reviewed by Pichler and Emmerstorfer-Augustin, 2018).

The Daum lab for example optimized the isolation of diverse cellular compartments, and then thoroughly analyzed the lipid profiles, e.g., of the plasma membrane (PM) (Grillitsch et al., 2014), peroxisomes (Wriessnegger et al., 2007), mitochondria (Wriessnegger et al., 2009), lipid droplets (Ivashov et al., 2012), and the endoplasmic reticulum (Klug et al., 2014). In all studies it was concluded that cellular membranes from *K. phaffii* are more similar to the membranes found in multicellular eukaryotes than those found in *S. cerevisiae*. For example, lipids found in *K. phaffii* harbor higher amounts of polyunsaturated fatty acids, while *S. cerevisiae* can only produce unsaturated and monounsaturated fatty acids. The presence of polyunsaturated fatty acids in the PM was linked to improved protein secretion (Wei et al., 2010), which may explain the pole position of *K. phaffii* as yeast protein secretion host. As expected, the PM of *K. phaffii* was shown to be enriched in ergosterol, although not to the same extend as in *S. cerevisiae* (Zinser et al., 1993). Plasma membranes from *K. phaffii* were also enriched in complex sphingolipids (glycosyl), inositol phosphorylceramides [(G)IPCs], over neutral sphingolipids, which accumulated in internal membranes (Grillitsch et al., 2014).

Due to their low abundance and highly diverse molecular structures, the analysis of sphingolipids is more complex than that of other lipids. Studies on *S. cerevisiae* gave important insights into sphingolipid metabolism and functions (Dickson, 2010), but *S. cerevisiae* is an exception among eukaryotic organisms because it contains only (G)IPCs. Most other fungi contain both (G)IPCs and glycosylceramides (GlcCers). Hence, *K. phaffii* is an attractive alternative model organism to investigate the biosynthesis of GlcCer, because it produces both sphingolipid classes (Michaelson et al., 2009; Ternes et al., 2011).

Lipid profiles of peroxisomes isolated from *K. phaffii* cells grown on methanol and oleic acid revealed that independent from the carbon source, phosphatidylcholine, and phosphatidylethanolamine were the major peroxisomal phospholipids (Wriessnegger et al., 2007). Cardiolipin was present in peroxisomal membranes at a substantial amount. Interestingly, growth on oleic acid changed fatty acid compositions of phospholipids extracted from total cells and peroxysomes. Phospholipids were shown to majorly incorporate oleic acid indicating that the fatty acid is not only utilized as carbon source but also as a direct building block for complex membrane lipids.

*K. phaffii* is also an exceptional well-suited host for the examination of lipid droplets. In general, the synthesis of non-polar lipids has been investigated in *K. phaffii* and contributing proteins have been identified (Ivashov et al., 2012). Different from *S. cerevisiae* and similar to *Y. lipolytica, K. phaffii* lipid droplets have a very high triacylgylercol to steryl ester ratio (Koch et al., 2014; Table 1).

## The Cell Wall

Cell walls comprise some 20–30% of the cell dry weight and are essential for the survival of fungal cells. Hence, cell wall perturbations and changes in cell wall morphology are efficiently transmitted to, and regulated by, the cell wall integrity (CWI) pathway (Levin, 2011). In *S. cerevisiae*, the response to cell wall stress signals is triggered by a family of cell-surface sensors, Wsc1, Wsc2, Wsc3, Mtl1, and Mid2, who activate the Pkc1-Mpk1 MAP kinase pathway. These sensors have highly conserved structural domains and consist of extracellular hyperglycosylated regions that function as mechanosensors, short single transmembrane domains, and flexible, cytosolic tails responsible for signal transduction and protein turnover (reviewed by Kock et al., 2015). Once cell wall sensors activate Rho1, which in turn activates protein kinase C (Pkc1), the signaling cascade is fired efficiently, resulting in signal transduction via several MAP kinases, and, finally, in hyperphosphorylation and nuclear localization of the terminal MAPK Slt2.

So far, several homologous proteins of the CWI pathway have been identified in *K. phaffii*. In 2001, the *K. phaffii* homolog of Slt2 was characterized and named Pim1 (*P. pastoris* cell integrity MAPK) (Cosano et al., 2001). Similar to what has been published for *Sc*Slt2, Pim1/Slt2 is dually phosphorylated, and thereby activated in response to heat stress, caffeine and agents that affect the integrity of the fungal cell wall. However, no link to any upstream signaling components has been examined in this study. A more recent publication characterized and investigated CWI sensors Wsc1, Wsc2, and Wsc3 in *K. phaffii* (Ohsawa et al., 2017). The study revealed a novel function of Wsc1 and Wsc3 in sensing and responding to external methanol levels. Additionally, the binding to Rom2, the down-stream Rho1 GDP-GTP nucleotide exchange factor, has been investigated. In another study, a methanol-induced upregulation of Pim1/Slt2 has been reported (Zhang et al., 2020). Cell wall integrity also explained the majority of variations among different *K. phaffii* strains (e.g., CBS7435 and GS115), impacting transformation efficiency, growth, methanol metabolism, and secretion of heterologous proteins (Brady et al., 2020).

In a screening assay searching for a super secretor strain, a mutation of *BGS13* (Beta-galactosidase supersecretion) was found to improve extracellular levels of a variety of secreted reporter proteins (Larsen et al., 2013). A BLAST search revealed that Bgs13 is the *K. phaffii* homolog of *S. cerevisiae* Pkc1. The role of Bgs13/Pkc1 has been further investigated by analyzing strains expressing the truncated variant of this essential protein (Naranjo et al., 2019). The mutant strain showed an abnormal localization of the Bgs13 variant and its cell wall suffered from inherent structural problems, which most probably promoted improved protein secretion.

## Vive La (Bio)Diversity: Concluding Remarks

**FIGURE 3:**
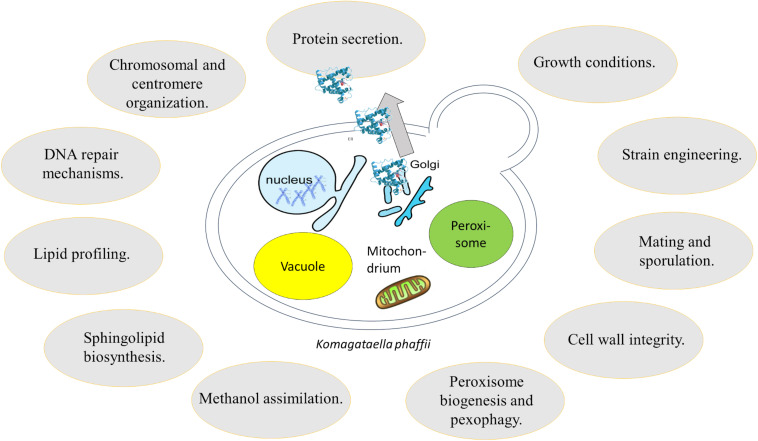
Overview of research fields addressed using *K. phaffii*.

As summarized aptly by Matthews and Vosshall, there are 10 essential steps to build a genetic model organism, including “learning how to work with the organism in the lab” and “develop precise mutagenesis for tagged mutants and gene replacement” (Matthews and Vosshall, 2020). *K. phaffii*, which has a strong track record as expression host in biotechnological applications, has mostly been in the focus of straightforward pathway engineering in order to leverage advantageous phenotypes. However, data obtained from next generation sequencing and system wide—omics studies in combination with advanced strain modification tools allow a better understanding of the unique physiology and metabolism of this yeast. Thereby, *K. phaffii* easily passed the first nine steps toward becoming a model system, and is currently tackling the last step, “grow a field of interesting questions using your new model organism.” One may ask now: why should we add additional species to the already existing, most common model systems? The answer is fairly simple. Evolution greatly diversified life on our planet, with many secrets yet to be revealed and many mechanisms yet to be discovered. This review highlights that the study of specialist species, such as *K. phaffii* gives new and important insights in diverse areas of biology (Figure 3). As shown numerous times, results produced with *K. phaffii* significantly differ from those obtained using *S. cerevisiae* (Table 1). Even though *S. cerevisiae* still is an essential key player in molecular biology and genetics, it is not a very good representative of yeast, since it has a rather atypical physiology compared to others. To summarize a few specifics, *S. cerevisiae* is a Crabtree-positive yeast, it prefers HR over NHEJ as DNA repair mechanism, and it did undergo whole genome duplication. Without any doubt, *S. cerevisiae* still offers many advantages including tools such as yeast two-hybrid screening assays (Ito et al., 2001), yeast oligo-mediated genome engineering (YOGE, a recombineering strategy) (Dicarlo et al., 2013) and the yeast deletion collection (Giaever and Nislow, 2014). While most of these tools are not feasible in *K. phaffii*, some alternative strategies are on the rise to fill this niche. Strategies like REMI which cause random integration and knockout of genes, will allow the performance of genome-wide loss or gain of function studies (Larsen et al., 2013). Another emerging strategy for the application of genome-wide screens is the application of CRISPR/Cas9 or CRISPRi. While the strategy has already widely been used and validated in mammalian studies, an application in *K. phaffii* is still missing. CRISPR-Cas9 can be used to randomly introduce an insertion or deletion in the genome, which can facilitate the screening for, and analysis of desirable phenotypes. Taken together, future research and engineering efforts will further leverage *K. phaffii* as powerful model organism to address a critical lack of fundamental biochemical information, maximize desired phenotypes, and increase productivity to reach industrially relevant production yields of new products.

## Author Contributions

LB, AR, LL, and AE-A drafted the manuscript. AE-A worked on all figures and tables and carefully reviewed the manuscript. All authors contributed to the article and approved the submitted version.

## Conflict of Interest

The authors declare that the research was conducted in the absence of any commercial or financial relationships that could be construed as a potential conflict of interest.

## Footnotes

1 https://www.validogen.com/unlock-pichia-technology
